# Use of a Si/CdTe Compton Camera for *In vivo* Real-Time Monitoring of Annihilation Gamma Rays Generated by Carbon Ion Beam Irradiation

**DOI:** 10.3389/fonc.2020.00635

**Published:** 2020-05-19

**Authors:** Shintaro Shiba, Raj Kumar Parajuli, Makoto Sakai, Takahiro Oike, Tatsuya Ohno, Takashi Nakano

**Affiliations:** ^1^Department of Radiation Oncology, Gunma University Graduate School of Medicine, Maebashi, Japan; ^2^Gunma University Heavy Ion Medical Center, Maebashi, Japan; ^3^Department of Molecular Imaging and Theranostics, National Institutes for Quantum and Radiological Science and Technology, Inage, Japan

**Keywords:** carbon ion beams, Compton camera, annihilation gamma-rays, adaptive therapy, irradiated site visualization, activated elements with annihilation gamma-ray transport visualization

## Abstract

The application of annihilation gamma-ray monitoring to the adaptive therapy of carbon ion radiotherapy (C-ion RT) requires identification of the peak intensity position and confirmation of activated elements with annihilation gamma-rays generated at the C-ion-irradiated site from those transported to unirradiated sites. Real-time monitoring of C-ion-induced annihilation gamma-rays was implemented using a Compton camera in a mouse model. An adult C57BL/6 mouse was anesthetized, and C-ion beams were directed into the abdomen at 1 × 10^9^ particles/s for 20 s. The 511 keV annihilation gamma-rays, generated by the interaction between the irradiated C-ion beam and the target mouse, were detected using a silicon/cadmium telluride (Si/CdTe) Compton camera for 20 min immediately after irradiation. The irradiated site and the peak intensity position of 511 keV gamma emissions due to C-ion beam irradiation on a mouse were observed at the abdomen of the mouse by developing Compton images. Moreover, the positron emitter transport was observed by evaluating the range of gamma-ray emission after the C-ion beam irradiation on the mouse. Our data suggest that by confirming the peak intensity and beam range of C-ion RT with Si/CdTe-based Compton camera, it would be possible to reduce the intra-fractional and inter-fractional dose distribution degradation. Therefore, the results of this study would contribute to the future development of adaptive therapy with C-ion RT for humans.

## Introduction

Compton cameras were originally developed in the field of astronomy to observe black-holes, supernovae, and the extreme universe (1, 2). A fundamental Compton camera consists of two types of position sensitive sub-detectors: scatterer and absorber. It can be used to take images of radioisotope (RI) distribution based on the kinematics of Compton scattering. Incident gamma-rays are scattered in the scatterer and are photo-absorbed in the absorber. From the detected energy—both in the scatterer and absorber—the scattered angle can be calculated as

(1) $$\begin{array}{r} \cos\theta =1-\frac{m_{e}c^{2}E_{1}}{E_{2}\left(E_{1}+E_{2}\right)}, \end{array}$$

where, m_e_c^2^ is the rest mass energy of an electron, *E*_1_ is the energy deposition in the scatterer, and *E*_2_ is the energy deposited in the absorber. Thus, the direction of the incident gamma-ray is restricted within a cone surface using the position and energy information from the two sub-detectors. Because a Compton camera does not require a mechanical collimator, it can be downsized to detect high energy gamma-rays more efficiently compared with conventional collimated cameras (3–5). A Compton camera is a compact (i.e., ~44.5 × 34.0 × 23.5 cm in size) device that can detect multiple radionuclides exhibiting a wide energy range (from a few hundred keV up to few MeV) simultaneously. These features indicate the device's potential for applications in medical imaging (6–13).

Carbon ion radiotherapy (C-ion RT) is an emerging cancer treatment modality (14–18). C-ion beams achieve a dose distribution superior to those of other radiotherapy modalities based on a distal tail-off of the Bragg peak and a sharp lateral penumbra (19–21). Although they can provide excellent dose localization at the target, treatment plans for C-ion RT are particularly sensitive to anatomical change because the range of a C-ion beam is determined by the tissue density along the path; therefore, subtle organ motions or setup error can significantly distort the intended dose distribution (22–25). Hence, achievement of adaptive therapy in C-ion RT is of utmost importance.

The detection of annihilation gamma-rays can be applied in C-ion RT verification, as positron emitters are generated by nuclear reactions in tissues irradiated with C-ions. Previous studies demonstrate a method for post-treatment verification of C-ion RT using auto-activation positron emission tomography (AAPET) that detects the 511 keV annihilation gamma-rays (26–28). However, AAPET has the disadvantage of large instrument size; thus, it is difficult to install an AAPET device in the treatment room, requiring patients to be transported from the treatment room for the imaging. This generates a time lag between the C-ion irradiation and AAPET imaging, which allows transport of the activated elements with annihilation gamma-rays to unirradiated tissues via biofluids (29) and results in blurring of the obtained image. Therefore, it is not advisable to use AAPET for C-ion RT verification, unless the device is sufficiently downsized for in-room installation. A Compton camera is capable of detecting the 511 keV annihilation gamma-rays and may easily be installed in the treatment room (30), suggesting its applicability in C-ion RT verification.

Therefore, by using a Compton camera, we demonstrate the first *in vivo* evidence of real-time monitoring of annihilation gamma-rays generated by C-ion irradiation. We further demonstrate the visualization of transported activated elements with annihilation gamma-rays from those generated at the irradiated site.

## Materials and Methods

### Compton Camera

We used a commercial Compton camera (ASTROCAM 7000HS, Mitsubishi Heavy Industries Ltd., Japan), which consists of eight layers of Si detectors in the scattering layer and four layers of CdTe detectors in the absorbing layer (31, 32). The Compton camera was initially developed to monitor environmental radiation following the accident of Fukushima Daiichi Power Plant based on astronomy technology (33). The area size of each detector was 50 × 50 mm. The thicknesses of the Si and CdTe layers were both 0.75 mm. The spacing between the layers of the Si and CdTe layers were 7.2 and 7.8 mm, respectively. The distance between the final layer of the Si detector and the first layer of the CdTe detector was 40 mm. The angular resolution measure (ARM) was 5.4° (full width half maximum) in angular resolution measure at 662 keV. The distance from the beam axis to the first Si detector in the scattering layers was 175 mm. It was not modified or specialized for this experiment. Further details are described elsewhere in previous studies (31, 32). The objects of the analysis in this study were the events detecting energy depositions in the Si detector and one of the CdTe detectors simultaneously (two-hit events). The energy window was set from 501 to 521 keV. The lists of two-hit event data, for which the total energy deposition in the Si and CdTe detectors was 501–521 keV, were read-out. The images were reconstructed by using the uniformly enlarged projection method. Gaussian distribution with ARM was adopted to spread the Compton rings (34).

### Carbon Ion Beams

We performed beam-monitoring experiments using C-ion beam at the Gunma University Heavy Ion Medical Center (31). A vertical beam-line C-ion pencil beam with a beam energy of 140 MeV/u was used in this experiment. The beam width was measured using Gafchromic film (EBT3, Ashland Inc., USA).

### Point Source Study

To validate the detecting point, we conducted imaging of the Na-22 point source (0.8 MBq; SKR8252, Eckert & Ziegler, Germany) with a diameter of 2 mm. First, the Na-22 point source was placed on a sample stage and measured; it was then placed 2 cm (equivalent to the height of the mouse) above the sample stage using an acrylic block and measured. The distance from the Na-22 point source to the first Si detector was 175 mm. The 511 keV gamma-ray emissions from the Na-22 RI were measured using the Compton camera for 3 min, and the measurement results were fit with a general parametric function (GPF) in equation (2) to obtain a point spread function (PSF). The GPF has a general form involving the Gaussian and Lorentzian functions by the parameter of *B*. As used below in Eq. (2), *V*_(*x*)_ represents the normalized pixel value at position *x*. The PSF is characterized by three characteristic parameters; *x*_0_, *A*, and *B*. A Gaussian function is the most common function to express PSFs. Unfortunately, however, the Gaussian function is not suitable, and GPF possesses better PSFs in Compton imaging than Gaussian, Lorentzian, and Voigt functions (35, 36).

(2) $$\begin{array}{r} V_{\left(x\right)}={\left(1+\frac{{\left(x-x_{0}\right)}^{2}}{A^{2}B}\right)}^{-B}, \end{array}$$

### Carbon Ion Beam Diameter Measurement

We performed C-ion beam irradiation on a Gafchromic film at 1 × 10^7^ particles/s for 1 min. Gafchromic films are radiation-sensitive films and are widely used in RT for dose verifications. Because of the dynamic range of the Gafchromic films, the beam flux was reduced from that used in the experiment of beam irradiation on a mouse. Flux-dependency of the beam-size was not observed. The irradiating beams cause a change in the color within the irradiated areas of the Gafchromic films indicating the shape and size of the beam. The resulting beam shape was digitalized using a scanner (Offirio ES-10000G, Epson, Japan) to evaluate the size of the ionized area. The image data were saved in TIFF format with 24-bit color, and only the red channel was analyzed. Finally, the beam size was confirmed using a Gaussian function fit to the measured result.

### Experiment of Carbon Ion Beam Irradiation on a Mouse

**Figure 1:**
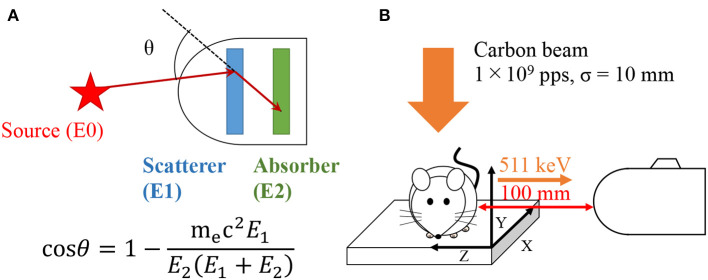
Schematic view of Compton camera **(A)** and experimental setup **(B)**.

We used an adult C57BL/6 mouse (Japan SLC Inc., Japan) for the present experiment. The mouse was anesthetized by intraperitoneal administration of 10% Somnopentyl, placed on the sample stage in a supine position, and immobilized with a thin holding fixture. The distance from the Compton camera to the center of abdomen in mouse was 100 mm (Figure 1). We performed C-ion beam irradiation on the abdomen of the mouse at 1 × 10^9^ particles/s for 20 s. Immediately after the C-ion beam irradiation, the 511 keV gamma-ray detection was performed for 20 min using the Compton camera. All animal experiments were performed with approval from the Animal Care and Experimentation Committee (Gunma University).

## Results

### Point Source Imaging

**Figure 2:**
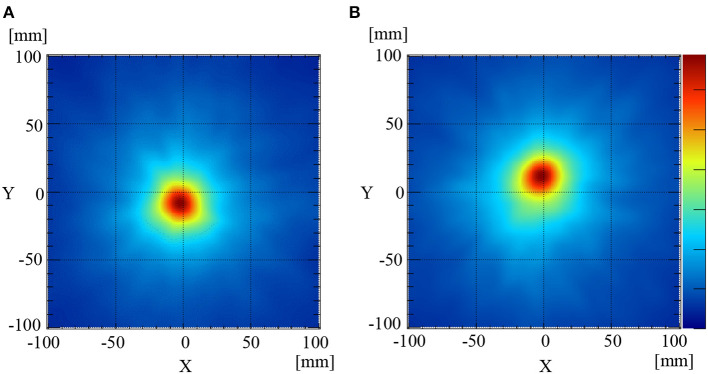
Compton images of the Na-22 point source placed at the[[Inline Image]] sample stage level **(A)** and 2 cm above the sample stage level **(B)**.

The 511 keV annihilation gamma-rays from the Na-22 point sources were detected using the Compton camera for 3 min. The numbers of selected Compton events used to reconstruct the image when the Na-22 was placed at the same level as the sample stage (mouse stage) and 2 cm above the sample stage were 1958 and 1901, respectively. The efficiency (selected events/incident gamma-rays) was ~0.05%, which was calculated from the setup geometry. Figures 2A,B illustrate the reconstructed back-projection images for the Na-22 point sources placed on the sample stage and 2 cm above the sample stage, respectively. GPFs were fit to the corresponding profiles pass through the peak position of the two images to estimate the PSFs. Using the newly obtained PSFs, the full width at half-maximums (FWHM) of the image of the Na-22 point sources placed on the sample stage (Figure 2A) and 2 cm above (Figure 2B) was determined to be 37.0 and 39.9 mm, respectively.

### Carbon Ion Beam Diameter Measurement

**Figure 3:**
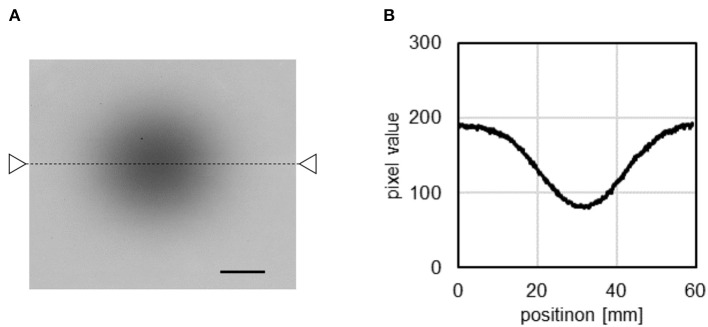
Result of the Gafchromic film measurement of the C-ion beams **(A)** and profile of the Gafchromic image **(B)**. Black bar = 1 cm.

Figure 3A illustrates the imaging of the C-ion beam irradiation with the Gafchromic film. The corresponding profile plot of the Gafchromic film image after beam irradiation (indicated by the triangles and dashed line in Figure 3A) is illustrated in Figure 3B. A Gaussian function was fitted to the plot profile along the x-projection around the center of the beam. The C-ion beam diameter (σ of the Gaussian distribution) was determined to be 10.2 mm in the direction of the x-axis (the horizontal direction in the Compton images). The 68% confidence interval was calculated to be less than 0.1 mm using the uncertainty in the film measurement of 3% (37, 38).

### Results of Carbon Ion Beam Irradiation of the Subject Mouse

**Figure 4:**
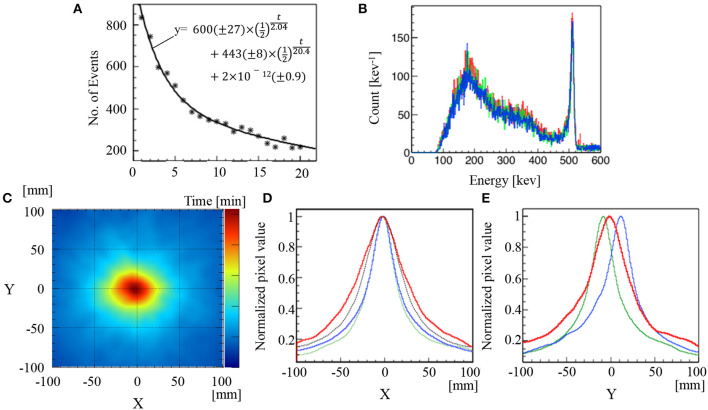
Results of C-ion beam irradiation on a mouse. Time course of Compton events and numbers in parentheses for fitting parameters are 68% confidence intervals **(A)**. Energy spectra of Na-22 or mouse-irradiation measurement **(B)**. Compton image **(C)**. Normalized profiles of x-projection at y = 0 (red line) **(D)** and normalized profiles of y-projection at x = 0 (red line) **(E)**. The dotted line in **(A)** represents a fitting curve following the equivalent equation, as illustrated in **(A)**. The red, green, and blue lines in **(B)** represent the corresponding spectrum of mouse and Na-22 point source images at the sample stage level and 2 cm above the sample stage level, respectively. The green lines and blue lines in **(D)** and **(E)** represent the corresponding profiles of Na-22 point source images at the sample stage level and 2 cm above the sample stage level, respectively. The black dotted line in **(D)** was the convolution of the PSF (FWHM = 40 mm) determined by the Na-22 image, and the beam profile (FWHM = 24 mm) determined by the Gafchromic film.

The 511 keV annihilation gamma-rays from a mouse irradiated with C-ion beams were detected using the Compton camera for 20 min immediately after C-ion beam irradiation for 20 s. The number of selected Compton events decreased from 833 to 220 events/min in this time period (Figure 4A). The total number of Compton events used to reconstruct the image after a 3-min measurement was 2,173. Figure 4B shows the energy spectra of two-hit data read out from the Compton camera, compared with those of the Na-22 measurements. The spectra are similar to each other. Figure 4C illustrates the reconstructed back-projection image of total composition for a mouse irradiated with C-ion beams. The x-projection profile map at y = 0 and the y-projection profile map at x = 0 of the corresponding back-projection reconstructed images depicted in Figure 4B are illustrated in Figure 4D (red line) and Figure 4E (red line), respectively. Additionally, the corresponding profiles of the x-projection at y = 0 and y-projection at x = 0 of the Na-22 point source images at the sample stage position are depicted in Figure 4D (green line) and Figure 4E (green line), respectively. Furthermore, the x-projection at y = 0 and y-projection at x = 0 for the Na-22 point source placed 2 cm above the sample stage position are depicted in Figure 4D (blue line) and Figure 4E (blue line), respectively. Upon evaluating the x-projection profile maps at y = 0, it was found that the peak projections were visualized at the same position when the Na-22 point source was placed at the sample stage level and when it was placed 2 cm above the sample stage level. This confirms that the target (mouse abdomen), the Na-22 point source placed at the stage level, and the Na-22 point source placed 2 cm above the stage level all lie along the vertical axis of beam delivery. For the y-projection profile map at x = 0, the peak projection due to C-ion beam irradiation of the mouse was visualized at a position between the peak projection profile of the Na-22 point source at the stage level and that of the Na-22 point source located 2 cm above the stage level. This confirms that both the Bragg peak position and the location of gamma-ray emission due to the C-ion beams are located well inside the target (mouse).

The FWHM of the range for detecting the generated region of the 511 keV gamma-ray after the C-ion beam irradiation was 72 mm. This diameter was markedly wider than the convolution of the C-ion beams evaluated by the Gafchromic film and the PSF evaluated by the Na-22 point source (black dotted line in Figure 4D). The FWHM of the convolution functions of the C-ion beam profile and the PSF, as can be observed in Figures 2A,B, was 55.2 and 58.2 mm, respectively.

## Discussion

We have demonstrated the use of a Compton camera for real-time monitoring of 511 keV annihilation gamma-ray emissions immediately after C-ion beam irradiation in a mouse. Unlike a PMMA or water phantom, the living body contains nitrogen, calcium, phosphorus, etc.; therefore, it may generate various types of isotopes and high energy gamma-rays. The high-density components scatter more gamma-rays. They could make considerably more noise events than does an acrylic phantom or water phantom. These noise effects can make it difficult to visualize gamma-ray emissions using a Compton camera. The C-ion beams mainly generate two β+ emitters, namely, C-11 (half-life: 20.4 min) and O-15 (half-life: 2.04 min) in PMMA, and the decay graph can be explained by the half-lives of the isotopes (Figure 4A) (39, 40). The composition of a mouse is slightly different from a PMMA. A considerable amount of positron emitters other than C-11 and O-15 could be generated. Some of them (e.g., O-14, N-13, F-17) have similar half-life times. Thus, further investigations are required to estimate the amount of positron emitters produced.

From the energy spectra of the Na-22 measurement and the mouse irradiation experiment, the shapes around 511 keV are similar. There would be scatter components of high-energy gamma-rays in the 501–521 keV energy window, however, the proportion is small. In this study, the impact of high energy gamma-rays is comparable to that of 1275 keV gamma-rays in the Na-22 measurement.

The peak projection visualized between the Na-22 point source at the sample stage level and 2 cm above the sample stage level indicates that the peak intensity was located in the abdomen of the mouse. Additionally, the reconstructed Compton image of 511 keV annihilation gamma-rays emitted after the C-ion beam irradiation on the mouse was relatively wider than was the image of the Gafchromic film generated by the C-ion beam irradiation. The beam profile appears to have the shape of a circle or an ellipse slightly elongated in the z-direction (vertical direction in Figure 3A). No position dependency was observed in the sigma estimation. The configuration of the body of the mouse is practically similar in the irradiation field (If the inhomogeneous of the body of the mouse strongly affected the distribution of the positron emitters, the peak position would change from the beam center and the distribution would be bilaterally asymmetric.) Thus, the distribution of the 511 keV source in the Compton image should be explainable in terms of the distribution of the C-ion beams and point spread function of the Compton image in the direction of the x-axis, if there is no diffusion in the body of the mouse. The PSF of the Compton image has position dependency. However, the impact of the difference of two Na-22 images on the FWHM of the convolution function is small (55.2 and 58.2 mm). There are some uncertainties in the FWHM estimation; the uncertainty of the pixel value of Compton image was not defined in the present study, however, the reproducibility of the PSF could be confirmed. By measuring the Na-22 on the sample stage three times, it was shown that the FWHM ± the standard deviation was 36.8 ± 0.2 mm (data not shown). Thus, the difference in the size of these Compton images would indicate that the positron emitters were transported via biofluids. These results can contribute to the future development of C-ion RT in clinical practice using Compton camera and radiological imaging for adaptive therapy. Unfortunately, the positron emitters would be spread throughout the body in the measurement time owing to the small size of the mouse. If the size of an object could be large and/or the efficiency could be increased, the kinetics would be observed.

The monitoring of the 511 keV annihilation gamma-rays was performed immediately after C-ion beam irradiation and not during the irradiation (on-beam condition), because a large number of secondary gamma-rays are generated during C-ion beam irradiation and this generates background noise affecting the detection of actual gamma-rays with the Compton camera. Moreover, the dead time of the Compton camera for gamma-ray detection in the on-beam condition is longer when compared to that in the off-beam condition. The projectile produces various secondary radiation, such as prompt gamma-rays and secondary-electron bremsstrahlungs (41–45). The detector dead-time caused by the measurement of the secondary radiations reduces the detection efficiency of the annihilation gamma-rays. The dead-time of the Compton camera is mainly caused by the trigger logic and the readout electronics because the Compton camera has to process a large amount of data (33, 46). Thus, the impact of dead-time for the Compton camera is larger than that for conventional gamma cameras. Sometimes, the high frequency of secondary radiations introduces random coincidence events (5, 47). High-energy gamma-rays could be detected by chance in the photo-peak energy window of 511 keV scattering in the target object and/or in the scatterer detector. In addition, MeV gamma-rays generate annihilation gamma-rays through pair productions, and these undesirable data decrease the quality of the Compton image. One of the measures used to reduce noise involves the synchronization of the Compton camera with the beam delivery. The C-ion beams generated by synchrotron are delivered with a profile of 20 pulses/min (a 1 s pulse is ON following a 2 s OFF period for accelerating) (48). By synchronizing detection with this beam period and monitoring the gamma-rays only during the OFF pulses (2 s period), one would suppress the influence of noises from secondary gamma-rays even during the on-beam periods.

The Compton camera has the potential for use in adaptive therapy with C-ion RT in clinical practice. If the 511 keV annihilation gamma-rays can be precisely monitored using the Compton camera immediately after C-ion RT, it is possible to reduce the inter-fractional dose distribution degradation by altering the subsequent treatment plan by comparing the dose distribution of the primary treatment plan with that of the plan on the actual day of treatment. Additionally, by confirming the beam range of C-ion RT and adjusting the beam energy, bolus thickness, or range shifter, it is possible to reduce intra-fractional dose distribution degradation. In this study, the peak intensities were not evaluated in a quantitative way. A quantitative analysis of Compton images is still at an early stage of development (49–51). Additionally, differentiating transported activated elements with annihilation gamma-rays from those generated at the irradiated site is not possible. It is of crucial importance to estimate the dose distribution and confirm the differentiation of annihilation gamma-rays; therefore, further investigations are required.

Several researchers have investigated whether AAPET is useful for treatment verification with proton beam therapy and C-ion RT and they have concluded that AAPET verification is useful and reliable (28, 29). However, owing to the large size of the instrumentation, current research with AAPET is mainly conducted with PET installed in a room separate from the irradiation room. Therefore, there occurs a time lag of ~10 min due to the transport of the patient from the irradiation room to the PET room. This time lag has a significant effect on the activated elements with annihilation gamma-rays transport and metabolic washouts and results in a lack of accuracy. Thus, OpenPET is being researched for the purpose of downsizing the device and detecting the annihilation gamma-rays during irradiation (52). However, in OpenPET, interference between the treatment beam axis and the detector lowers the detection accuracy. Moreover, the cost and size of the OpenPET constitute disadvantages. In contrast, Compton cameras are compact and do not generate interference with the treatment beam axis.

It is easier to conduct basic research using our portable Compton camera because there are relatively fewer restrictions on the experimental location and layout. Additionally, this approach is expected to aid clinical trials in humans because it has no negative effect on patients, in contrast to the radiation exposure in clinical trials using computed tomography. At present, the spatial resolution of our Compton camera is not sufficient to develop a system for human imaging. However, a spatial resolution of 5 mm or less can be achieved by optimizing the distance between the beam axis and the detector, enhancing the hardware by adding/removing detectors, or enhancing the application-specific integrated circuit (ASIC) of the Compton camera. A high performance ASIC can reduce the accidental coincidence event and maintain the detection efficiency when the spatial resolution is increased.

## Conclusions

We have demonstrated real-time monitoring of 511 keV annihilation gamma-ray emissions immediately after C-ion beam irradiation of a mouse and have visualized the peak intensity of the gamma-ray events and activated elements with annihilation gamma-rays transport in the mouse abdomen using a Si/CdTe-based Compton camera. By confirming the peak intensity and beam range of C-ion RT using a Si/CdTe-based Compton camera, it is possible to reduce the intra-fractional and inter-fractional dose distribution degradation. Because the gamma-ray emission site can trace the Bragg peak location (25), we were able to confirm that the Bragg peak lies within the mouse abdomen in this study. Additionally, the activated elements with annihilation gamma-rays transport, as evaluated from the Compton images, reveals that the activated elements with annihilation gamma-rays transport within the mouse abdomen was affected by biofluids. These results can contribute to the future development of C-ion RT and are expected to aid in human clinical trials by facilitating adaptive therapy and allowing accurate irradiation in response to anatomical changes.

## Data Availability Statement

All datasets generated for this study are included in the article/supplementary material.

## Ethics Statement

The animal study was reviewed and approved by The Animal Care and Experimentation Committee (Gunma University).

## Author Contributions

SS, RP, MS, and TOi: conceptualization, methodology, validation, formal analysis, investigation, and supervision. RP, MS, and TOi: resources. RP and MS: data curation. SS and RP: writing-original draft preparation. SS, MS and TOi: writing-review and editing. TN: visualization. MS: project administration. TOh and TN: funding acquisition.

## Conflict of Interest

The authors declare that the research was conducted in the absence of any commercial or financial relationships that could be construed as a potential conflict of interest.

## References

[1] Takahashi T Kokubun M Mitsuda K Kelley RL Ohashi T Aharonian F Hitomi (ASTRO-H) X-ray astronomy satellite. J Astron Telesc Instrum Syst. (2018) 4:2 10.1117/1.JATIS.4.2.021402

[2] Schoenfelder V Aarts H Bennett K Boer H Clear J Collmar W Instrument description and performance of the imaging gamma ray telescope COMPTEL aboard the Compton Gamma Ray Observatory. Astrophys J Suppl Ser. (1993) 86:657 10.1086/191794

[3] Han L Clinthorne NH Performance Evaluation of Compton Based Camera for High Energy Gamma Ray Imaging. IEEE Nuclear Science Symposium Conference Record (2005). p. 2561–5.

[4] Fontana M Ley J.-L Testa É Versatile Compton camera for high-energy gamma Rays: Monte Carlo comparison with anger camera for medical imaging. Acta Physica Polonica Series B. (2017) 48:1639 10.5506/APhysPolB.48.1639

[5] Fontana M Dauvergne D Létang JM Ley JL Testa É. Compton camera study for high efficiency SPECT and benchmark with Anger system. Phys Med Biol. (2017) 62:8794. 10.1088/1361-6560/aa926a 28994664

[6] Kishimoto A Kataoka J Taya T Tagawa L Mochizuki S Ohsuka S. First demonstration of multi-color 3-D *in vivo* imaging using ultra-compact Compton camera. Sci Rep. (2017) 7:2110. 10.1038/s41598-017-02377-w 28522868 PMC5437019

[7] Munekane M Motomura S Kamino S Ueda M Haba H Yoshikawa Y. Visualization of biodistribution of Zn complex with antidiabetic activity using semiconductor Compton camera GREI. Biochem Biophys Rep. (2016) 5:211–215. 10.1016/j.bbrep.2015.12.004 28955826 PMC5600336

[8] Nakano T Sakai M Torikai K Suzuki Y Noda SE Yamaguchi M Imaging of < sup>99m < /sup>Tc-DMSA and < sup>18 < /sup>F-FDG in humans using a Si/CdTe Compton camera. Phys Med Biol. (2019) 10.1088/1361-6560/ab33d8 31323647

[9] Sakai M Yamaguchi M Nagao Y Kawachi N Kikuchi M Torikai K *In vivo* simultaneous imaging with (99m)Tc and (18)F using a Compton camera. Phys Med Biol. (2018) 63:205006 10.1088/1361-6560/aae1d1 30222127

[10] Suzuki Y Yamaguchi M Odaka H Shimada H Yoshida Y Torikai K. Three-dimensional and multienergy gamma ray simultaneous imaging by using a Si/CdTe Compton camera. Radiology. (2013) 267:941–7. 10.1148/radiol.13121194 23418002

[11] McCleskey M Kaye W Mackin D Beddar S He Z Polf J Evaluation of a multistage CdZnTe Compton camera for prompt γ imaging for proton therapy. Nucl Instrum Methods Phys Res A. (2015) 785:163–9. 10.1016/j.nima.2015.02.030

[12] Howalt OS Kuvvetli I Budtz-JØrgensen C Zoglauer A Evaluation of a Compton camera concept using the 3D CdZnTe drift strip detectors. J Instrum. (2019) 14:01020 10.1088/1748-0221/14/01/C01020

[13] Schoene S An image reconstruction framework and camera prototype aimed for Compton imaging for *in-vivo* dosimetry of therapeutic ion beams. IEEE Trans Radiat Plasma Med Sci. (2017) 1:96–107. 10.1109/TNS.2016.2623220

[14] Kamada T Tsujii H Blakely EA Debus J De Neve W Durante M. Carbon ion radiotherapy in Japan: An assessment of 20 years of clinical experience. Lancet Oncol. (2015) 16:e93–e100. 10.1016/S1470-2045(14)70412-7 25638685

[15] Shiba S Abe T Shibuya K Katoh H Koyama Y Shimada H Carbon ion radiotherapy for 80 years or older patients with hepatocellular carcinoma. BMC Cancer. (2017) 17:721 10.1186/s12885-017-3724-4 29115938 PMC5678597

[16] Ohno T Noda SE Murata K Yoshimoto Y Okonogi N Ando K. Phase I study of carbon ion radiotherapy and image-guided brachytherapy for locally advanced cervical cancer. Cancers Basel. (2018) 10:338. 10.3390/cancers10090338 30231543 PMC6162662

[17] Shibuya K Ohno T Katoh H Okamoto M Shiba S Koyama Y A feasibility study of high-dose hypofractionated carbon ion radiation therapy using four fractions for localized hepatocellular carcinoma measuring 3cm or larger. Radiother Oncol. (2018) 132:230–5. 10.1016/j.radonc.2018.10.009 30366726

[18] Shiba S Shibuya K Katoh H Kaminuma T Miyazaki M Kakizaki S. A comparison of carbon ion radiotherapy and transarterial chemoembolization treatment outcomes for single hepatocellular carcinoma: a propensity score matching study. Radiat Oncol. (2019) 14:137. 10.1186/s13014-019-1347-4 31375120 PMC6679447

[19] Kanai T Endo M Minohara S Miyahara N Koyama-ito H Tomura H. Biophysical characteristics of HIMAC clinical irradiation system for heavy ion radiation therapy. Int J Radiat Oncol Biol Phys. (1999) 44:201–10. 10.1016/S0360-3016(98)00544-6 10219815

[20] Nakano T Suzuki Y Ohno T Kato S Suzuki M Morita S. Carbon beam therapy overcomes the radiation resistance of uterine cervical cancer originating from hypoxia. Clin Cancer Res. (2006) 12(7 Pt 1):2185–90. 10.1158/1078-0432.CCR-05-1907 16609033

[21] Tsujii H Kamada T Shirai T Noda K Tsuji H Karasawa K (editors) Carbon Ion Radiotherapy. Tokyo: Springer; Science & BusinessMedia (2013) 10.1007/978-4-431-54457-9

[22] Abe S Kubota Y Shibuya K Koyama Y Abe T Ohno T. Fiducial marker matching versus vertebral body matching: Dosimetric impact of patient positioning in carbon ion radiotherapy for primary hepatic cancer. Phys Med. (2017) 33:114–20. 10.1016/j.ejmp.2016.12.018 28057427

[23] Irie D Saitoh JI Shirai K Abe T Kubota Y Sakai M. Verification of dose distribution in carbon ion radiation therapy for stage I lung cancer. Int J Radiat Oncol Biol Phys. (2016) 96:1117–23. 10.1016/j.ijrobp.2016.09.002 27869084

[24] Sakai M Kubota Y Saitoh JI Irie D Shirai K Okada R Robustness of patient positioning for inter-fractional error in carbon ion radiotherapy for stage I lung cancer: Bone matching versus tumor matching. Radiother Oncol. (2018) 129:95–100. 10.1016/j.radonc.2017.10.003 29100701

[25] Shiba S Saitoh JI Irie D Shirai K Abe T Kubota Y. Potential pitfalls of a fiducial marker-matching technique in carbon ion radiotherapy for lung cancer. Anticancer Res. (2017) 37:5673–80. 10.21873/anticanres.12003 28982885

[26] Zhang J Lu Y Hsi W Zhang J Sheng Y Shi L. Evaluation of proton therapy accuracy using a PMMA phantom and PET prediction module. Front Oncol. (2018) 8:523. 10.3389/fonc.2018.00523 30483477 PMC6243057

[27] Parodi K Enghardt W. Potential application of PET in quality assurance of proton therapy. Phys Med Biol. (2000) 45:N151–6. 10.1088/0031-9155/45/11/403 11098922

[28] Bauer J Unholtz D Sommerer F Kurz C Haberer T Herfarth K. Implementation and initial clinical experience of offline PET/CT-based verification of scanned carbon ion treatment. Radiother Oncol. (2013) 107:218–26. 10.1016/j.radonc.2013.02.018 23647759

[29] Shimizu M Sasaki R Miyawaki D Nishimura H Demizu Y Akagi T. Physiologic reactions after proton beam therapy in patients with prostate cancer: significance of urinary autoactivation. Int J Radiat Oncol Biol Phys. (2009) 75:580–6. 10.1016/j.ijrobp.2009.02.085 19735884

[30] Parajuli RK Sakai M Kada W Torikai K Kikuchi M Arakawa K. Annihilation gamma imaging for carbon ion beam range monitoring using Si/CdTe Compton camera. Phys Med Biol. (2019) 64:055003. 10.1088/1361-6560/ab00b2 30669125

[31] Matsuura D Genba K Kuroda Y Ikebuchi H Tomonaka T “ASTROCAM 7000HS” radioactive substance visualization camera. mitsubishi heavy industries. Tech Rev. (2014) 51:68–75.

[32] Takeda S Harayama A Ichinohea Y Odaka H Watanabe S Takahashi T A portable Si/CdTe Compton camera and its applications to the visualization of radioactive substances. NIM-A. (2015) 787:207–11. 10.1016/j.nima.2014.11.119

[33] Watanabe S Tajima H Fukazawa Y Ichinohe Y Takedaa S Enoto T The Si/CdTe semiconductor Compton camera of the ASTRO-H Soft Gamma-ray Detector (SGD). NIM-A. (2014) 765:192–201. 10.1016/j.nima.2014.05.127

[34] Takeda S Odaka H Ishikawa S Watanabe S Aono H Takahashi T Demonstration of *in-vivo* multi-probe tracker based on a Si/CdTe semiconductor Compton camera. IEEE Transac Nucl Sci. (2012) 59:70–6. 10.1109/TNS.2011.2178432

[35] Kim SM Seo H Park JH Kim CH Lee CS Lee SJ. Resolution recovery reconstruction for a Compton camera. Phys Med Biol. (2013) 58:2823–40. 10.1088/0031-9155/58/9/2823 23563165

[36] Sakai M. Effect of number of views on cross-sectional Compton imaging: A fundamental study with backprojection. Phys Med. (2018) 56:1–9 10.1016/j.ejmp.2018.11.006 30527083

[37] Marroquin EY Herrera González JA Camacho López MA Evaluation of the uncertainty in an EBT3 film dosimetry system utilizing net optical density. J Appl Clin Med Phys. (2016) 8:466–81. 10.1120/jacmp.v17i5.6262 PMC5874103 27685125

[38] Sorriaux J Kacperek A Rossomme S Leea JA Bertrandd D Vynckiera S. Evaluation of Gafchromic® EBT3 films characteristics in therapy photon, electron and proton beams. Physica Medica. (2013) 29:599–606. 10.1016/j.ejmp.2012.10.001 23107430

[39] Bongrand A Busato E Force P Martin F Montarou G. Use of short-lived positron emitters for in-beam and real-time β+ range monitoring in proton therapy. Phys Med. (2020) 69:248–55. 10.1016/j.ejmp.2019.12.015 31918377

[40] Pshenichnov I Mishustin I Greiner W. Distributions of positron-emitting nuclei in proton and carbon-ion therapy studied with GEANT4., Phys Med Biol. (2006) 51:6099–112. 10.1088/0031-9155/51/23/011 17110773

[41] Yamaguchi M Nagao Y Ando K Yamamoto S Sakai M Parajuli RK. Imaging of monochromatic beams by measuring secondary electron bremsstrahlung for carbon-ion therapy using a pinhole x-ray camera. Phys Med Biol. (2018) 63:045016. 10.1088/1361-6560/aaa17c 29235991

[42] Ishii K Kamiya M Sera K Morita S Tawara H Directional anisotropy of secondary-electron bremsstrahlung induced by proton bombardment of thin solid target. Phys Rev A. (1977) 15:2126 10.1103/PhysRevA.15.2126

[43] Krimmer J Dauvergne D Létang JM Testa E Prompt-gamma monitoring in hadrontherapy: a review. NIM-B. (2018) 878:58–73. 10.1016/j.nima.2017.07.063

[44] Testa E Bajard M Chevallier M Dauvergne D le Foulher F Freud N Dose profile monitoring with carbon ions by means of prompt-gamma measurements. NIM-B. (2009) 267:993–6. 10.1016/j.nimb.2009.02.031

[45] Richard M.-H Chevallier M Dauvergne D Freud N Henriquet P le Foulher F Design guidelines for a double scattering Compton camera for prompt-γ imaging during ion beam therapy: a Monte Carlo simulation study. IEEE Trans Nucl Sci. (2011) 58:87–94. 10.1109/TNS.2010.2076303

[46] Uhlmann N Wolfel S Pauli J Anton G 3D-position-sensitive compact scintillation detector as absorber for a Compton-camera. IEEE Trans Nucl Sci. (2005) 52:606–11. 10.1109/TNS.2005.851454

[47] Ortega PG Espallardo IT Böhlen TT Cerutti F Chin MPW Ferrari A Noise Evaluation of Prompt-Gamma Technique for Proton-Therapy Range Verification Using a Compton Camera. 2013. IEEE Nuclear Science Symposium and Medical Imaging Conference (2013). p. 1–7

[48] Ohno T Kanai T Yamada S Yusa K Tashiro M Shimada H Carbon ion radiotherapy at the Gunma University Heavy Ion Medical Center: new facility setup. Cancers. (2011) 3:4046–4060. 10.3390/cancers3044046 24213124 PMC3763409

[49] Sakai Kubota Y Parajuli RK Kikuchi M Arakawa K Nakano T. Compton imaging with 99mTc for human imaging. Sci Rep. (2019) 9:12906. 10.1038/s41598-019-49130-z 31501461 PMC6733951

[50] Andreyev A Sitek A Celler A. Fast image reconstruction for Compton camera using stochastic origin ensemble approach. Med Phys. (2011) 38:429–438. 10.1118/1.3528170 21361211

[51] Taya T Kataoka J Kishimoto A Tagawa L Mochizuki S Toshito T Optimization and verification of image reconstruction for a Compton camera towards application as an on-line monitor for particle therapy. J Instrum. (2017) 12:P07015 10.1088/1748-0221/12/07/P07015

[52] Tashima H Yoshida E Inadama N Nishikido F Nakajima Y Wakizaka H. Development of a small single-ring OpenPET prototype with a novel transformable architecture. Phys Med Biol. (2016) 61:1795–809. 10.1088/0031-9155/61/4/1795 26854528

